# Associations of Lifestyle Patterns With Overweight and Depressive Symptoms Among United States Emerging Adults With Different Employment Statuses

**DOI:** 10.3389/ijph.2023.1606451

**Published:** 2023-11-21

**Authors:** Qian-Wen Xie, Xu Li Fan, Xiangyan Luo, Jieling Chen

**Affiliations:** ^1^ Department of Social Welfare and Risk Management, School of Public Affairs, Zhejiang University, Hangzhou, China; ^2^ Research Center for Common Prosperity, Future Regional Development Laboratory, Innovation Center of Yangtze River Delta, Zhejiang University, Jiaxing, China; ^3^ Center of Social Welfare and Governance, Zhejiang University, Hangzhou, China; ^4^ School of Nursing, Sun Yat-sen University, Guangzhou, China

**Keywords:** lifestyle, depression, overweight, emerging adult, latent class analysis

## Abstract

**Objective:** To identify lifestyle patterns in emerging adults and examine the association of lifestyle patterns with overweight and depression.

**Methods:** Data was from the National Health and Nutrition Examination Survey between 2011 and 2018. A latent class analysis (LCA) was conducted with 2,268 US emerging adults based on sedentary behavior, moderate-to-vigorous physical activity, diet, sleep, alcohol drinking, and cigarette smoking. The associations of lifestyle groups with overweight and depression were examined by logistic regression and were further stratified by employment status.

**Results:** The LCA results favored a four-class solution: “unhealthy but non-substance use” (59%), “healthy but sleepless and drinking” (12%), “unhealthy lifestyle” (15%), and “healthy but sedentary” group (14%). Compared to the “unhealthy lifestyle” group, participants in the “unhealthy but non-substance use” (coef. = −1.44, SE = 0.27; OR = 0.40, 95% CI [0.26, 0.61]), “healthy but sleepless and drinking” (coef. = −1.49, SE = 0.35; OR = 0.38, 95% CI [0.20, 0.72]), and “healthy but sedentary” (coef. = −1.97, SE = 0.34; OR = 0.29, 95% CI [0.14, 0.57]) groups had lower depression severity and reported fewer depressive symptoms. Moreover, lifestyle groups and health outcomes showed different relationships among employed and unemployed participants.

**Conclusion:** This study found that the combinations of lifestyle behaviors had synergistic effects on mental health, and such effects differed by employment status.

## Introduction

Emerging adulthood, which includes individuals aged between 18 and 25 years old, is a distinct developmental period when lifelong habits are formed and many unhealthy behaviors are developed, profoundly influencing health over the rest of the life course [1, 2]. In this transition to adulthood, people are faced with the confusion of multiple identities and roles and the formulation of intimate relationships [2, 3], resulting in dramatic changes in personal behaviors along with health issues [1]. In fact, the prevalence of several unhealthy behaviors, such as binge drinking and cigarette use, peaks not during adolescence but during emerging adulthood [1]. Young people at this stage of life are gaining weight faster than any other age group [4]. In the US, over half of young people at this age are either overweight or obese [5]. Moreover, the prevalence of depression among emerging adults has been soaring in recent years, with more than a quarter of adolescents having been diagnosed with depression at some point in their life [6]. Therefore, it is essential to focus on the health status of this unique population and identify risk factors to develop effective interventions and address these health challenges.

The term “lifestyle” includes the characteristics and manifestations of people’s daily life [7]. Healthy lifestyle behaviors are important contributors to the prevention and management of obesity and depression for emerging adults [8–14], and those behaviors include more physical activity, less sedentary behavior, sufficient sleep, a balanced and nutritious diet, no or moderate drinking, and no smoking [15–19]. However, most of the studies evaluating emerging adults’ lifestyles used university students as samples, lacking exploration of the broader emerging adult population. The only study that used a broad emerging adult sample only examined the lifestyles of those aged 22 years [9]. The small sample size and nonnationally representative population included in that study also limited the generalizability of the results. Moreover, the wide use of university student samples also excludes workers in emerging adulthood. Apart from the transition to adulthood, these young workers also experience the transition to being a member of society and the adjustment issue in the work environment [20]. These unique problems may lead to more serious health conditions for them [21]; however, whether there are associations between lifestyle and health outcomes among employed emerging adults remains unknown.

According to problem-behavior theory [22] and health lifestyle theory [23], health behaviors have conventionality, and all healthy or unhealthy lifestyle behaviors tend to cluster together based on a person’s social status and class [24–26]. Moreover, the combinations of lifestyle behaviors have synergistic effects, which might be greater than the additive effects of each behavior [27, 28]. As the interest in clustered health behaviors is increasing, latent class analysis (LCA), a person-centered approach, has been introduced in lifestyle research [29]. Compared with traditional variable-centered approaches, this method treats the individual as a whole and detects potential lifestyle groups within the population [30]. Moreover, LCA minimizes subjectivity by using statistical metrics to indicate the best model fit and number of classes [29]. To date, a large body of extant studies have remained focused on traditional variable-centered approaches, and for those using LCA, nearly all of them have relied on non-representative populations [15, 29, 31, 32].

With a nationally representative sample of US emerging adults aged 18–25 years, the current study aimed to [1] identify potential lifestyle groups among emerging adults with the use of LCA [2]; assess whether the identified lifestyle groups were associated with overweight and depression; and [3] examine whether the associations of lifestyle with overweight and depression were different between employed and unemployed participants. It was hypothesized that distinct lifestyle groups existed among US emerging adults and that the groups characterized by healthy lifestyle behaviors were associated with better health outcomes, which varied by employment status.

## Methods

### Study Population

The National Health and Nutrition Examination Survey (NHANES) is a large, population-based survey conducted by the National Center for Health Statistics (NCHS) in the United States each year that assesses the lifestyle and health condition for all noninstitutionalized civilian US population. It employs a systematic and multistep probability sampling design to draw a nationally representative household sample. Each 2 years cycle data are combined to create a dataset. The data utilized in the current study were obtained through in-person interviews and physician-performed medical examinations. In this study, we used four cycles of datasets spanning from 2011 to 2018. This specific time frame was set due to the limited information on the lifestyle behaviors of our target population in the datasets collected prior to 2011 and subsequent to 2018. The survey protocol was approved by the NCHS Ethics Review Board. More details of the study design and data collection can be found on the NCHS’s website [33, 34]. Out of all young adults aged 18–24 years old, we excluded participants who had missing data on the lifestyle behavior variables (814 participants) and outcome variables (26 participants), resulting in a total sample of 2,268 participants for the analyses of the current study. The characteristics of participants with complete data on lifestyle behaviors and outcome variables and those with missing data are shown in Supplementary Table S1 in the Appendix.

### Measurements

#### Lifestyle Behaviors

Data on all lifestyle behaviors were obtained from sample in-person questionnaires and 24 h dietary recalls. Then, we dichotomized six lifestyle variables, including sedentary behavior, moderate-to-vigorous physical activity (MVPA), diet, sleep, alcohol drinking and cigarette smoking. Sedentary behavior was assessed by the time spent sitting per day, and sitting for more than 7.5 h a day was defined as unhealthy (coded as 0) according to previous studies [19, 35]. MVPA was self-reported and measured by the average number of minutes engaged in leisure-time moderate and vigorous activities per day. According to recommendations of the World Health Organization (WHO), at least 150 min of moderate-intensity physical activity, 75 min of vigorous-intensity physical activity or an equivalent combination of moderate- and vigorous-intensity physical activity per week was defined as healthy (coded as 1) [19]. Diet was assessed by the 2015 Healthy Eating Index (HEI-2015) scores through 13 components in the 24 h dietary recalls, including total fruits, total vegetables, whole grains, dairy, sodium, added sugars, etc. [36]. HEI-2015 scores, ranging from 0 to 100, reflected overall dietary quality, and individuals with scores in the top two quintiles were considered healthy (coded as 1) [37]. Sleep was reported by participants using the average number of hours of sleep per day regardless of weekdays or weekends, and sleep between 6 and 8 h was considered healthy (coded as 1) [38]. Alcohol drinking was identified by the average number of drinks on the days in which an alcoholic beverage was consumed, and a healthy level was defined as the consumption of two drinks or fewer for men and one drink or fewer for women (a drink = a 12 oz beer, a 5 oz glass of wine, or 1.5 oz of liquor), according to the Dietary Guidelines for Americans (coded as 1) [39]. Never smoking was regarded as healthy (coded as 1), which was identified by a response of “No” to the question, “Have you smoked at least 100 cigarettes in your entire life?” [40].

#### Overweight

Young adults’ height and weight were measured through physician-performed medical examinations to calculate body mass index (BMI). Moreover, to indicate the weight status of emerging adults more directly, we also recoded 0 as under and normal weight (BMI <25) and 1 as overweight and obese (BMI ≥25) [41].

#### Depressive Symptoms

Depressive symptoms were assessed using the Patient Health Questionnaire-9 (PHQ-9), with summed scores ranging from 0–27. The Cronbach’s alpha of the PHQ-9 in this study was 0.80, indicating good internal consistency. The severity of depression was categorized into 5 levels: 1 = minimal (“0–4”), 2 = mild (“5–9”), 3 = moderate (“10–14”), 4 = moderately severe (“15–19”), and 5 = severe (“20–27”) [42]. Moreover, we combined the categories of moderate and severe and defined those participants as having depressive symptoms (coded as 1), and we combined the categories of minimal and mild and defined those participants as no depressive symptoms (coded as 0) [42, 43].

#### Employment Status

Work status was a dichotomous variable and was defined by the type of work performed in the last week; the responses of “employed at a job or business” and “with a job or business but not at work” were considered employed (coded as 0), and “looking for work” and “unemployed” were considered unemployed (coded as 1) [44].

#### Covariates

The following variables were included as covariates due to their potential confounding effects. Sociodemographic characteristics included age, sex (1 = male; 2 = female), race/ethnicity (1 = non-Hispanic White; 2 = non-Hispanic Black; 3 = others), and immigrant status (1 = native; 2 = immigrant). Moreover, household income was measured by the poverty income ratio (PIR), a ratio of income to the federal poverty threshold, and it was categorized into three levels: 1 = high (>3.5), 2 = middle (1.3–3.5), and 3 = low (≤1.3) [45]. The education level and household reference person’s education level were categorized into three levels: 1 = less than high school degree, 2 = high school graduate or some college degree, and 3 = college graduate or above [40]. The insurance status was reported by participants (1 = having health insurance; 2 = no health insurance) [46]. To avoid the influence of changes over time, we also adjusted the NHANES cycle (1 = 2011–2012 cycle, 2 = 2013–2014 cycle, 3 = 2015–2016 cycle, 4 = 2017–2018 cycle) as a covariate.

### Data Analysis

First, LCA was deployed to identify the underlying groups that existed within young adults based on the six lifestyle behaviors. A succession of models using the selected lifestyle behaviors was tested with 2–6 classes. The optimal number of latent classes was determined by model fit, class proportions, and the interpretability of the results. The model fit was judged by several statistical indices: the Akaike information criterion (AIC), the Bayesian information criterion (BIC), the adjusted BIC (aBIC), the parametric bootstrapped likelihood ratio test (BLRT) and the interpretability of the results. Smaller AIC, BIC, and aBIC values indicated better model fit, and significant BLRT values indicated that the k class model had a better fit than the k-1 class model. After the latent classes were determined, all young adults were assigned to one of the identified lifestyle groups based on the highest posterior probability of membership. The identified lifestyle groups were compared using ANOVAs for continuous variables and chi-squared tests for categorical variables. The associations of lifestyle groups with overweight and depression were then analyzed using linear regression for BMI and depression severity, and logistic regression for weight status and depressive symptoms. Furthermore, we examined the associations of lifestyle groups with overweight and depression stratified by employment status to explore whether employed or unemployed status affects the relationship between lifestyle and health.

A sensitivity analysis was performed by repeating LCA models with data that further removed missing values on the covariates. Then, we further adjusted for a health-related variable, i.e., general health status (1 = excellent or good; 2 = fair or poor), in the association analyses to test the robustness of the results.

The LCA was conducted using Mplus version 8.3. All other analyses were conducted using R version 4.1.1. Two-sided *p* values less than 0.05 were considered to indicate statistical significance.

## Results

### Latent Class Analysis of Lifestyle Behaviors

Table 1 displays the LCA model with 2–6 classes against the fit indicators. From the 5-class model onward, the BLRT values were insignificant, and the smallest class proportion was less than 5%. Therefore, the 4-class solution was favored due to its interpretability, and its AIC and aBIC values were the smallest, indicating a good fit.

**TABLE 1:** Model fit indices for latent class analysis with 2–6 classes. United states, 2011–2018.

Class	AIC	BIC	aBIC	BLRT	Proportion
2	17,668.93	17,743.38	17,702.07	0.00	0.53/0.47
3	17,637.90	17,752.44	17,695.86	0.00	0.12/0.71/0.16
4	17,627.03	17,781.65	17,688.89	0.00	0.59/0.12/0.15/0.14
5	17,631.51	17,826.22	17,718.19	1.00	0.13/0.11/0.08/0.66/0.01
6	17,638.44	17,873.24	17,742.97	1.00	0.01/0.09/0.09/0.05/0.69/0.08

Note: AIC, Akaike’s information criterion; BIC, Bayesian information criterion; aBIC, Adjusted Bayesian information criterion; BLRT, bootstrapped likelihood ratio test.

**FIGURE 1:**
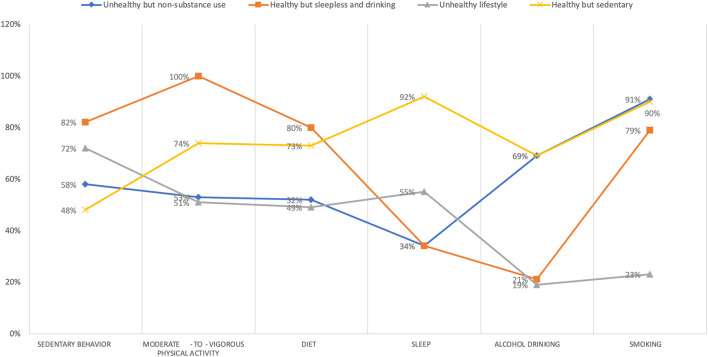
Item-response probabilities of healthy lifestyle behaviors by the four latent class groups, United States, 2011–2018. Note: “Unhealthy but non-substance use” group (Class 1) represented 59% of the sample (*n* = 1,339). “Healthy but sleepless and drinking” group (Class 2) accounted for 12% of the full sample (*n* = 280). “Unhealthy lifestyle” group (Class 3) represented 15% of the sample (*n* = 333). “Healthy but sedentary” group (Class 4) represented 14% of the sample (*n* = 316).

Classes were labeled based on their distinguishing features (see Figure 1). Young adults in Class 1 and Class 3 both had a relatively lower probability of healthy lifestyle behaviors, including adequate MVPA (53% and 51.95%), less sedentary behavior (58% and 72%), nutritious diet (52% and 49%), and healthy sleep (34% and 55%), but they differed in substance use behaviors. Therefore, Class 1 (*n* = 1,339, 59%) was identified as the “unhealthy but non-substance use” group with the highest probability of non-drinkers or moderate drinkers (69%) and non-smokers (91%). On the other hand, with the lowest probability of no or moderate drinking (81%) and no smoking (77%), Class 3 (n = 333, 15%) was defined as the “unhealthy lifestyle” group since it had a lower probability of almost all healthy lifestyle behaviors. Moreover, Class 2 (*n* = 280, 12%) was labeled as the “healthy but sleepless and drinking” group, which had the highest probability of adequate physical activity (100%), less sedentary behavior (82%), and healthy diet (80%), but also a great proportion of sleepless people (66%) and excessive drinkers (79%). Class 4 (*n* = 316, 14%), with almost all lifestyle behaviors relatively healthy except for sedentary behaviors (48%), was identified as the “healthy but sedentary” group.

### Descriptive Statistics by Lifestyle Groups

As shown in Table 2, participants (N = 2,268) were aged 18–24 years old (mean = 20.87, standard deviation [SD] = 2.04). Of all young adults, 50.40% were males, 31.35% were non-Hispanic White, 17.55% were immigrants, 16.71% met the criteria of high-income households, and a majority (63.7%) were employed. For overweight and depression, the average BMI value and depression severity of all young adults were 26.91 (SD = 7.31) and 1.37 (SD = 0.73), respectively. A total of 50.93% (*n* = 1,155) fell into overweight or obese status, and 8.02% (*n* = 182) reported having depressive symptoms. Young adults in different lifestyle groups significantly differed on all variables except family income. The “healthy but sleepless and drinking” group had significantly more employed young adults, while the “unhealthy but non-substance use” group had more non-workers. In addition, the “unhealthy lifestyle” group had a significantly higher BMI value, more severe depression, and a greater proportion of overweight or obese individuals with depressive symptoms than the other three groups.

**TABLE 2:** Descriptive statistics of sample participants, NHANES, United states, 2011–2018.

	Total (*n* = 2,268)	Unhealthy but non-substance use (*n* = 1,339, 59%)	Healthy but sleepless and drinking (*n* = 280, 12%)	Unhealthy lifestyle (*n* = 333, 15%)	Healthy but sedentary (*n* = 316, 14%)	F value/χ^2^
Age*	20.87 ± 2.04	20.62 ± 2.07	21.16 ± 1.89	21.60 ± 1.83	20.88 ± 2.07	F = 23.60 ***
Sex						**χ** ^**2**^ = 21.30 ***
Male	1,143 (50.40%)	626 (46.75%)	144 (51.43%)	198 (59.46%)	175 (55.38%)	
Female	1,125 (49.60%)	713 (53.25%)	136 (48.57%)	135 (40.54%)	141 (44.62%)	
Race/ethnicity						**χ** ^**2**^ = 62.20 ***
Non-Hispanic White	711 (31.35%)	358 (26.74%)	106 (37.86%)	153 (45.95%)	94 (29.75%)	
Non-Hispanic Black	543 (23.94%)	367 (27.41%)	44 (15.71%)	57 (17.12%)	75 (23.73%)	
Others	1,014 (44.71%)	614 (45.86%)	130 (46.43%)	123 (36.94%)	147 (46.52%)	
Immigrant status						**χ** ^**2**^ = 35.60 ***
Native	1,869 (82.41%)	1,097 (81.93%)	233 (83.21%)	309 (92.79%)	240 (75.95%)	
Immigrant	398 (17.55%)	241 (18.00%)	57 (20.36%)	24 (7.21%)	76 (24.05%)	
Household income						**χ** ^**2**^ = 8.21
High	379 (16.71%)	215 (16.06%)	53 (18.93%)	46 (13.81%)	65 (20.57%)	
Middle	729 (32.14%)	432 (32.26%)	91 (32.50%)	110 (33.03%)	96 (30.38%)	
Low	943 (41.58%)	547 (40.85%)	109 (38.93%)	157 (47.15%)	130 (41.14%)	
Education level						**χ** ^**2**^ = 55.00 ***
Less than high school degree	449 (19.80%)	270 (20.16%)	30 (10.71%)	92 (27.63%)	57 (18.04%)	
High school graduate or some college degree	1,448 (63.84%)	849 (63.41%)	202 (72.14%)	215 (64.56%)	182 (57.59%)	
College graduate or above	371 (16.36%)	220 (16.43%)	48 (17.14%)	26 (7.81%)	77 (24.37%)	
Household reference person’s education level						**χ** ^**2**^ = 45.10 ***
Less than high school degree	422 (18.61%)	258 (19.27%)	44 (15.71%)	72 (21.62%)	48 (15.19%)	
High school graduate or some college degree	1,306 (57.58%)	775 (57.88%)	162 (57.86%)	207 (62.16%)	162 (51.27%)	
College graduate or above	375 (16.53%)	207 (15.46%)	50 (17.86%)	30 (9.01%)	88 (27.85%)	
Health insurance status						**χ** ^**2**^ = 27.00 ***
Having health insurance	1,655 (72.97%)	996 (74.38%)	213 (76.07%)	205 (61.56%)	241 (76.27%)	
No health insurance	604 (26.63%)	337 (25.17%)	67 (23.93%)	127 (38.14%)	73 (23.10%)	
Employment status						**χ** ^**2**^ = 16.50 **
Employed	1,344 (59.26%)	752 (56.16%)	191 (68.21%)	207 (62.16%)	194 (61.39%)	
Unemployed	923 (40.70%)	587 (43.84%)	89 (31.79%)	125 (37.54%)	122 (38.61%)	
BMI*	26.91 ± 7.31	27.19 ± 7.65	25.90 ± 6.19	27.63 ± 7.43	25.84 ± 6.44	F = 5.82 ***
Weight status						**χ** ^**2**^ = 15.80 **
Overweight or obese	1,155 (50.93%)	696 (51.98%)	128 (45.71%)	192 (57.66%)	139 (43.99%)	
Normal or underweight	1,113 (49.07%)	643 (48.02%)	152 (54.29%)	141 (42.34%)	177 (56.01%)	
Depression severity*	1.37 ± 0.73	1.36 ± 0.73	1.32 ± 0.66	1.59 ± 0.88	1.23 ± 0.58	F = 14.50 ***
The presence of depressive symptoms						**χ** ^**2**^ = 35.00 ***
Having depressive symptoms	182 (8.02%)	101 (7.54%)	16 (5.71%)	52 (15.62%)	13 (4.11%)	
No depressive symptoms	2,086 (91.98%)	1,238 (92.46%)	264 (94.29%)	281 (84.38%)	303 (95.89%)	

Note: *Mean ± standard deviation; *p < 0.05, **p < 0.01, ***p < 0.001.

### Associations of Lifestyle Groups With Overweight and Depression

Among US emerging adults, lifestyle groups were associated with depressive symptoms (see Table 3). Compared to the “unhealthy lifestyle” group, participants in other lifestyle groups had less severe depression and were less likely to have depressive symptoms. The “unhealthy but non-substance use” group, the “healthy but sleepless and drinking” group, and the “healthy but sedentary” group were associated with a decreased depression severity of −1.44 (standard error [SE] = 0.27, *p* < 0.001), −1.49 (SE = 0.35, *p* < 0.001), and −1.97 (SE = 0.34, *p* < 0.001), respectively. Moreover, the odds of having depressive symptoms for these three groups were 60% (odds ratio [OR] = 0.40, 95% CI [0.26, 0.61], *p* < 0.001), 62% (OR = 0.38, 95% CI [0.20, 0.72], *p* < 0.001), and 71% (OR = 0.29, 95% CI [0.14, 0.57], *p* < 0.001) lower than those of the “unhealthy lifestyle” group, respectively. We did not find significant associations between lifestyle groups and overweight.

**TABLE 3:** Associations of lifestyle groups with overweight and depression. United states, 2011–2018.

	BMI		Weight status		Depression severity		Depressive symptoms	
	Coef.	SE	OR	95% CI	Coef.	SE	OR	95% CI
Unhealthy but non-substance use	0.02	0.50	0.94	(0.71, 1.24)	−1.44***	0.27	0.40***	(0.26, 0.61)
Healthy but sleepless and drinking	−1.14	0.65	0.72	(0.50, 1.03)	−1.49***	0.35	0.38***	(0.20, 0.72)
Healthy but sedentary	−0.68	0.63	0.78	(0.55, 1.11)	−1.97***	0.34	0.29***	(0.14, 0.57)

Note: Reference: “Unhealthy lifestyle” group; Coef., coefficient; SE, standard error; OR, odds ratio; CI, confidence interval; *p < 0.05, **p < 0.01, ***p < 0.001.

### Associations of Lifestyle Groups With Overweight and Depression Stratified by Employment Statuses

The variation in employment status in the associations of lifestyle groups with overweight and depression was further examined, and the results were slightly different from the pooled analyses (see Table 4). For emerging adults at work, the only significant association was between lifestyle groups and depression severity. Compared to the “unhealthy lifestyle” group, employed participants in two non-substance use groups, the “unhealthy but non-substance use” group (coefficient [Coef. ] = −0.13, SE = 0.06, *p* < 0.05) and the “healthy but sedentary” group (Coef. = −0.18, SE = 0.08, *p* < 0.05) had less severe depression. Similarly, unemployed emerging adults in the “unhealthy but non-substance use” group (Coef. = -0.43, SE = 0.08, *p* < 0.001; OR = 0.28, 95% CI [0.15, 0.55], *p* < 0.001), the “healthy but sleepless and drinking” group (Coef. = -0.45, SE = 0.11, *p* < 0.001; OR = 0.22, 95% CI [0.07, 0.63], *p* < 0.01), and the “healthy but sedentary” group (Coef. = -0.51, SE = 0.11, *p* < 0.001; OR = 0.20, 95% CI [0.07, 0.59], *p* < 0.01) also had less severe depression and were less likely to report depressive symptoms than those in the “unhealthy lifestyle” group. Of note, compared to the “unhealthy lifestyle” group, the “healthy but sleepless and drinking” unemployed participants were less likely to fall into overweight or obese status (OR = 0.46, 95% CI [0.24, 1.88], *p* < 0.05).

**TABLE 4:** Associations of lifestyle groups with overweight and depression stratified by employment status. United states, 2011–2018.

		BMI		Weight status		Depression severity		Depressive symptoms	
		Coef.	SE	OR	95% CI	Coef.	SE	OR	95% CI
Employed	Unhealthy but non-substance use	0.14	0.61	0.96	(0.66, 1.38)	−0.13*	0.06	0.55	(0.30, 1.01)
	Healthy but sleepless and drinking	−0.59	0.77	0.87	(0.55, 1.37)	−0.14	0.08	0.60	(0.27, 1.35)
	Healthy but sedentary	−0.22	0.76	0.76	(0.48, 1.19)	−0.18*	0.08	0.41	(0.16, 1.03)
Unemployed	Unhealthy but non-substance use	−0.23	0.85	0.90	(0.57, 1.42)	−0.43***	0.08	0.28***	(0.15, 0.55)
	Healthy but sleepless and drinking	−2.24	1.19	0.46*	(0.24, 0.87)	−0.45***	0.11	0.22**	(0.07, 0.63)
	Healthy but sedentary	−1.42	1.10	0.80	(0.45, 1.44)	−0.51***	0.11	0.20**	(0.07, 0.59)

Note: Reference: “Unhealthy lifestyle” group; Coef., coefficient; SE, standard error; OR, odds ratio; CI, confidence interval; *p < 0.05, **p < 0.01, ***p < 0.001.

### Results of Sensitivity Analyses

When the LCA model was applied to the data that further excluded missing values on covariates (*n* = 1,921), it also favored a four-class pattern that was similar to the results in the main analyses (see Supplementary Table S2 and Supplementary Figure S1 in the Appendix). Moreover, the associations of lifestyle groups with health outcomes were not materially changed after further adjusting for general health status (see Supplementary Tables S3, S4 in the Appendix).

## Discussion

Emerging adulthood is a phase of life connecting childhood and adolescence with adulthood, in which an individual’s lifestyle behaviors radically change [1, 2]. With a nationally representative sample, the current study found that lifestyle behaviors were clustered among US emerging adults aged 18–25 years and identified four distinct lifestyle groups: “unhealthy but non-substance use” group (Class 1), “healthy but sleepless and drinking” group (Class 2), “unhealthy lifestyle” group (Class 3), and “healthy but sedentary” group (Class 4).

To our knowledge, this study was the first to use a broad and representative sample of an emerging adult population. In line with previous studies [47–51], we found a group with a high probability of all unhealthy behaviors, namely, the “unhealthy lifestyle” group, comprising 15% of the overall sample. The largest group we found was the “unhealthy but non-substance use” group. These two groups had relatively more unhealthy lifestyle behaviors than the other two groups, accounting for approximately 75% of the total sample, indicating that overall, healthy lifestyle factors among US emerging adults need to be improved. On the other hand, unlike former studies, a group characterized by a large number of excessive drinkers but also a large number of non-smokers was identified, that is the “healthy but sleepless and drinking” group; thus, alcohol use and tobacco use do not necessarily cooccur. In fact, according to the report by the Centers for Disease Control and Prevention (CDC), binge drinking (≥5 drinks for men, ≥4 drinks for women) is most common in emerging adults in the US, with a percentage of 21% [52]; in contrast, cigarette smoking is lowest among people aged 18–24 years, with only 5.3% [53]. In particular, we discovered a lifestyle group, the “healthy but sedentary” group, that was healthy in all lifestyle behaviors except sedentariness. Consistent with the results of previous research from different countries [54–56], this meant that no typical “healthy” group was found among emerging adults. In our sample, all four groups were characterized by at least one unhealthy behavior, re-emphasizing the importance of improving lifestyle for emerging adults.

These unhealthy lifestyle factors have affected the health of emerging adults in the US [10]. In our study, more than half of the participants fell into overweight or obese status, and nearly 10% reported depressive symptoms; both were prominently higher than the statistics of overall US adults [57, 58]. Using the “unhealthy lifestyle” group as the reference, we found that lifestyle groups were associated with depressive symptoms. Consistent with previous studies using variable-centered approaches [32, 59, 60], groups with more healthy lifestyle behaviors had lower depression severity and reported fewer depressive symptoms. Moreover, the innovative and striking aspect of this study lies in the synergistic effect observed from the association coefficients of various combinations of lifestyle behaviors. More specifically, compared to the “unhealthy lifestyle” group, the extent of reduced depression severity and reduced number of people having depressive symptoms in the “healthy but sedentary” group characterized by five healthy behaviors was greater than the “healthy but sleepless and drinking (four healthy behaviors) and the “unhealthy but non-substance use” group (two healthy behaviors), extending the evidence for the synergistic effect of clustered lifestyle to the field of mental health.

Of note, while BMI and weight status significantly varied across lifestyle groups, the significance did not persist when examining their associations with lifestyle. First, it could be partly explained by the severe weight condition among US emerging adults [57]. The average BMI values for both the overall sample and all lifestyle groups, regardless of how many health behaviors were exhibited, were greater than 25 (the cutoff of overweight or obese status). The high prevalence of obesity, coupled with unhealthy lifestyles, has been linked to an increased risk of cardiovascular disease, higher overall mortality rates, and various other health problems [37]. Second, from a biological perspective, puberty at an earlier age is a major risk factor for adult obesity; specifically, earlier pubertal maturation can lead to adiposity in emerging adulthood [61, 62]. Third, another explanation for this contradiction with previous studies was probably the use of a broad sample of emerging adults, rather than only university students [21, 63].

Therefore, we further investigated whether employment status made a difference in the relationship between lifestyle and health outcomes. We found that among non-workers, being in the “healthy but sleepless and drinking” group was associated with less overweight or obesity. Therefore, the inclusion of workers in the sample attenuated the relationship between lifestyle and overweight or obesity, corroborating the above explanation. Similar results have been found in previous research in that the association between lifestyle and BMI was not significant among workers [64], but the reasons behind this phenomenon remain obscure and need to be further explored. With respect to mental health, the relationship between lifestyle and depressive symptoms was much closer for unemployed participants than for those at work. Among non-workers, all groups had significantly lower depression compared to the “unhealthy lifestyle” group, and the coefficients and significance among unemployed emerging adults were much greater than those at work. For participants at work, with a new identity that is completely different from their identity as “student” which they are accustomed to, they face adjustment problems and experience more pressure from work; these issues are also contributing factors to mental illnesses, which might diminish the role of lifestyle in mental health [2, 3, 20]. The only significant association between lifestyle group and mental health we found was among the two non-substance groups, i.e., the “unhealthy but non-substance use” group and the “healthy but sedentary” group. Past research has suggested that high substance use, including alcohol and tobacco use, among young workers is detrimental to mental health [65, 66], indicating an area of focus for emerging-adult interventions.

Considering these findings, efforts to improve the lifestyles and health of emerging adults should focus on two key areas. First, approaches that promote multiple healthy lifestyle behaviors to take advantage of their synergistic effects on health remain a priority. There are several emerging adult-specific lifestyle interventions, including physical activity [67–69], nutrition [70–72], sleep [73, 74], and substance use [75], but most of those are still single- or dual-behavior medication trials that neglect the clustering of lifestyles. Moreover, despite the existing consensus-based recommendations for healthy lifestyle behaviors [19, 76, 77], to date, the optimal delivery pattern and combination strategy for multiple lifestyle behavior interventions are not known. Our findings extended the evidence that approaches to promote multiple healthy lifestyle behaviors should be supported and pointed to possible combinations of different lifestyle behaviors for future research as well.

Second, it is imperative to design more tailored lifestyle interventions for emerging adults with different identities. According to Erikson’s stages of development [3], young people at this stage are faced with shifting identities, and the different identities might have a persistent and far-reaching impact on their behaviors [1, 78]. However, most extant studies on emerging adult-specific lifestyle interventions merely consider the campus environment when carrying out community-based practices [70, 74]. Workers in emerging adulthood have always been neglected. Our findings that the associations of lifestyle with overweight and depression differed between employed and unemployed participants provide initial evidence that targeted strategies of lifestyle modification for employed and unemployed emerging adults should be tested. Specifically, for those at work, workplace-based lifestyle interventions have showed long-term effectiveness in tackling their overweight and obesity and improving their mental health [79]. Implementing community-based lifestyle interventions that involve the family might also be an effective approach in improving their overall health. Furthermore, it is crucial to prioritize the regulation of substance use, particularly the excessive alcohol consumption, among this group of young adults. On the other hand, for unemployed emerging adults, adopting a healthy lifestyle is more important for maintaining good mental health. Since a significant number of unemployed individuals at this age are university students, it is crucial to include peer support as a means of encouraging healthy behaviors, and implementing campus-based lifestyle interventions may be necessary to promote their overall wellbeing.

### Limitations

We must consider limitations. The first is the cross-sectional nature of the NHANES data, precluding the determination of causality in the findings. Second, self-reported measures were used to assess lifestyle behaviors and depression, which may be prone to recall bias; for example, nutrition intake might be underreported, and physical activity might be overreported [80]. Therefore, the results of the current study must be interpreted with caution, and research with objective measures, such as accelerometry, is needed. Third, we were unable to explore the lifestyle and health status of emerging adults during COVID-19 due to constraints on data availability. It would be intriguing to investigate the differences between pre-pandemic and pandemic periods in future studies. Fourth, due to the lack of information regarding study status in the data, we did not further distinguish those who are employed students and those who are unemployed non-students and did not consider more types of employment (e.g., full-time and part-time job). It should also be note that using a cut-off point of 100 cigarettes to define smokers might induce bias, as it might not accurately capture new smokers who have not yet reached this threshold, and might still categorize ex-smokers as smokers. In future studies, more heterogeneity among the employed and unemployed populations and among smokers and quitters needs to be further explored.

### Conclusion

In conclusion, with the use of a representative and broad sample of US emerging adults, this study indicated that lifestyle groups were associated with mental health, and such associations markedly differed by employment status. These findings supported the improvement in population health by promoting a combination of multiple healthy lifestyle behaviors and, more importantly, by carrying out targeted lifestyle interventions for employed and unemployed individuals.
