# Supplementary material for: Associations of Lifestyle Patterns With Overweight and Depressive Symptoms Among United States Emerging Adults With Different Employment Statuses
Source: Int J Public Health. 2023 Nov 21;68:1606451. doi: 10.3389/ijph.2023.1606451 (PMC10696088; doi:10.3389/ijph.2023.1606451)
Supplement: Supplementary file 1 [file DataSheet1.docx]

**eTable 1. Comparison of sociodemographic characteristics between data with missing values and without missing values on lifestyle behaviors and health outcomes.**

|  | **With missing values** (n=3,108) | **Without missing values** (n=2,268) | **P value** |
| --- | --- | --- | --- |
| Age^*^ | 20.66 ± 2.08 | 20.87 ± 2.04 | 0.30 |
| Sex |  |  | 0.70 |
| Male | 1,549 (50.16%) | 1,143 (50.40%) |  |
| Femlae | 1,559 (49.84%) | 1,125 (49.60%) |  |
| Race/ethnicity |  |  | 0.40 |
| Non-Hispanic White | 923 (29.70%) | 711 (31.35%) |  |
| Non-Hispanic Black | 756 (24.32%) | 543 (23.94%) |  |
| Others | 1,429 (45.98%) | 1,014 (44.71%) |  |
| Immigrant status |  |  | 0.30 |
| Native | 2,528 (81.34%) | 1,869 (82.41%) |  |
| Immigrant | 579 (18.66%) | 398 (17.55%) |  |
| Household income |  |  | 0.30 |
| High | 473 (15.22%) | 379 (16.71%) |  |
| Middle | 960 (30.89%) | 729 (32.14%) |  |
| Low | 1,329 (42.76%) | 943 (41.58%) |  |
| Education level |  |  | 0.01 |
| Less than high school degree | 699 (22.49%) | 449 (19.80%) |  |
| High school graduate or some college degree | 1,863 (59.94%) | 1,448 (63.84%) |  |
| College graduate or above | 545 (17.54%) | 371 (16.36%) |  |
| Household reference person's education level |  |  | 0.20 |
| Less than high school degree | 634 (20.40%) | 422 (18.61%) |  |
| High school graduate or some college degree | 1,761 (56.66%) | 1,306 (57.58%) |  |
| College graduate or above | 473 (15.22%) | 375 (16.53%) |  |
| Health insurance status |  |  | 0.90 |
| Having health insurance | 2,261 (72.75%) | 1,655 (72.97%) |  |
| No health insurance | 831 (26.74%) | 604 (26.63%) |  |
| Employment status |  |  | 0.08 |
| Employed | 1,766 (56.82%) | 1,344 (59.26%) |  |
| Unemployed | 1,341 (43.18%) | 923 (40.70%) |  |

Note: *Mean ± standard deviation.

**eTable 2. Model fit indices for latent class analysis with 2-6 classes in the sensitivity analysis.**

| **Class** | **AIC** | **BIC** | **aBIC** | **BLRT** | **Proportion** |
| --- | --- | --- | --- | --- | --- |
| 2 | 14,966.43 | 15,038.72 | 14,997.42 | 0.00 | 0.49/0.51 |
| 3 | 14,942.68 | 15,053.89 | 14,990.35 | 0.00 | 0.21/0.15/0.64 |
| 4 | 14,934.32 | 15,084.45 | 14,998.68 | 0.00 | 0.66/0.06/0.20/0.09 |
| 5 | 14,935.57 | 15,124.63 | 15,016.61 | 0.25 | 0.10/0.06/0.15/0.61/0.08 |
| 6 | 14,943.46 | 15,171.44 | 15,041.18 | 1.00 | 0.02/0.06/0.14/0.10/0.67/0.02 |

Note: AIC: Akaike's information criterion; BIC: Bayesian information criterion; aBIC: Adjusted Bayesian information criterion; BLRT: bootstrapped likelihood ratio test.

**eFig 1. Item-response probabilities of healthy lifestyle behaviors by the four latent class groups in the sensitivity analysis.**


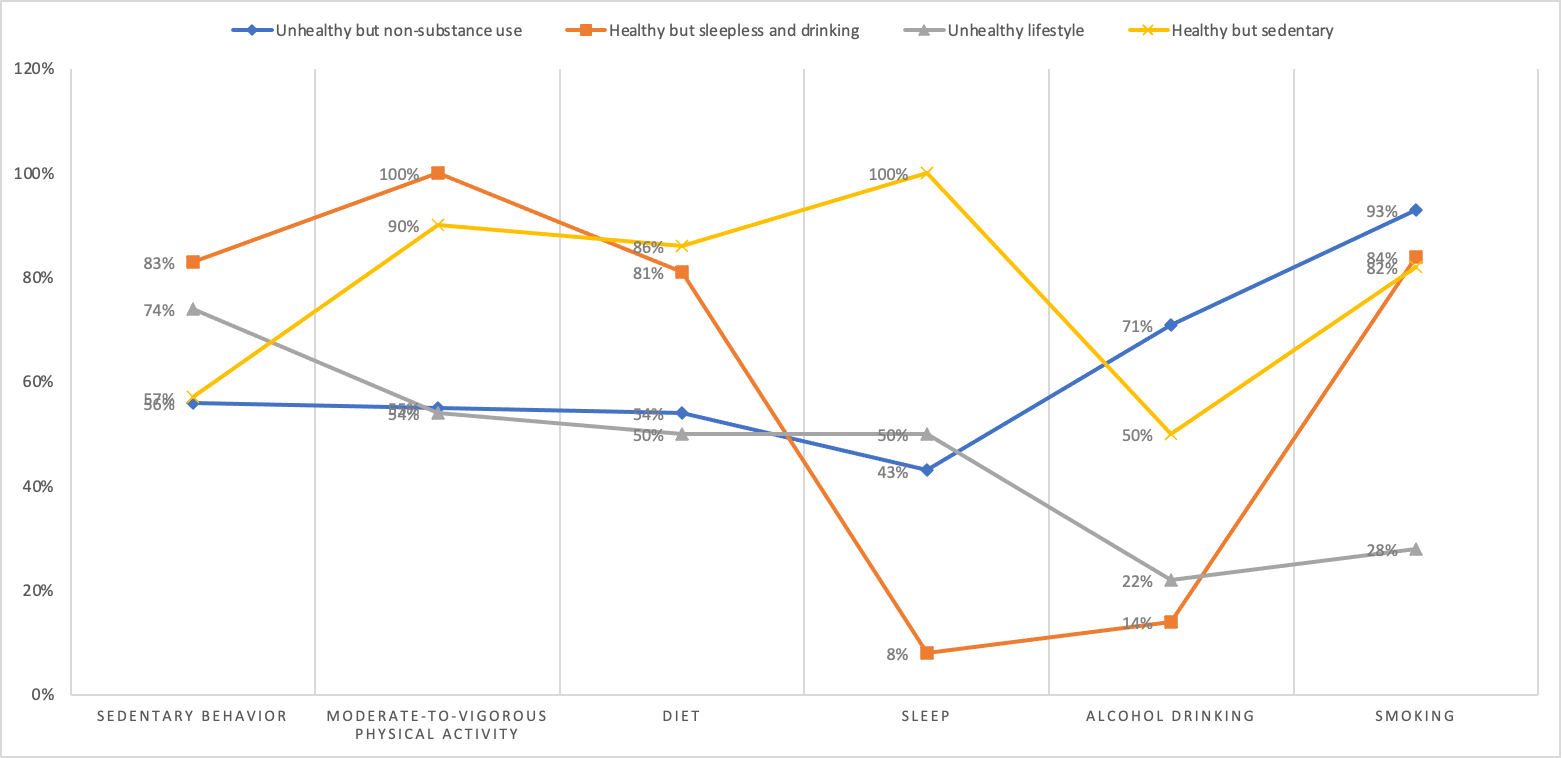


Note: “Unhealthy but non-substance use” group (Class 1) represented 66% of the sample (n=1,268). “Healthy but sleepless and drinking” group (Class 2) accounted for 6% of the full sample (n=115). “Unhealthy lifestyle” group (Class 3) represented 20% of the sample (n=384). “Healthy but sedentary” group (Class 4) represented 9% of the sample (n=173).

**eTable 3. Associations of lifestyle groups with overweight and depression in the sensitivity analysis.**

|  | **BMI** | | **Weight status** | | **Depression severity** | | **Depressive symptoms** | |
| --- | --- | --- | --- | --- | --- | --- | --- | --- |
|  | **Coef.** | **SE** | **OR** | **95% CI** | **Coef.** | **SE** | **OR** | **95% CI** |
| Unhealthy but non-substance use | 0.17 | 0.49 | 0.97 | (0.73, 1.28) | -0.23*** | 0.05 | 0.41*** | (0.27, 0.63) |
| Healthy but sleepless and drinking | -0.83 | 0.64 | 0.77 | (0.53, 1.10) | -0.23*** | 0.06 | 0.43*** | (0.22, 0.80) |
| Healthy but sedentary | -0.38 | 0.62 | 0.82 | (0.58, 1.18) | -0.29*** | 0.06 | 0.31*** | (0.15, 0.60) |

Note: Reference: “Unhealthy lifestyle” group; Coef.: coefficient; SE: standard error; OR: odds ratio; CI: confidence interval; *p<0.05, **p<0.01, ***p<0.001.

eTable 4. Associations of lifestyle groups with overweight and depression stratified by employment status in the sensitivity analysis.

|  |  | **BMI** | | **Weight status** | | **Depression severity** | | **Depressive symptoms** | |
| --- | --- | --- | --- | --- | --- | --- | --- | --- | --- |
|  |  | **Coef.** | **SE** | **OR** | **95% CI** | **Coef.** | **SE** | **OR** | **95% CI** |
| Employed | Unhealthy but non-substance use | 0.25 | 0.61 | 0.97 | (0.67, 1.41) | -0.12 | 0.06 | 0.57 | (0.31, 1.06) |
|  | Healthy but sleepless and drinking | -0.38 | 0.76 | 0.91 | (0.57, 1.43) | -0.12 | 0.08 | 0.67 | (0.29, 1.47) |
|  | Healthy but sedentary | -0.04 | 0.76 | 0.78 | (0.49, 1.23) | -0.16* | 0.08 | 0.44 | (0.16, 1.06) |
| Unemployed | Unhealthy but non-substance use | -0.08 | 0.84 | 0.94 | (0.59, 1.48) | -0.41*** | 0.08 | 0.27*** | (0.14, 0.52) |
|  | Healthy but sleepless and drinking | -1.78 | 1.17 | 0.50* | (0.26, 0.96) | -0.39*** | 0.11 | 0.25* | (0.08, 0.71) |
|  | Healthy but sedentary | -1.00 | 1.09 | 0.88 | (0.45, 1.44) | -0.46*** | 0.11 | 0.21** | (0.06, 0.60) |

Note: Reference: “Unhealthy lifestyle” group; Coef.: coefficient; SE: standard error; OR: odds ratio; CI: confidence interval; *p<0.05, **p<0.01, ***p<0.001.
